# Classification of Malicious URLs Using Machine Learning

**DOI:** 10.3390/s23187760

**Published:** 2023-09-08

**Authors:** Shayan Abad, Hassan Gholamy, Mohammad Aslani

**Affiliations:** Department of Computer and Geo-Spatial Sciences, University of Gävle, 801 76 Gävle, Sweden; shayan.abad@prevas.se (S.A.); zizou.hassan22@gmail.com (H.G.)

**Keywords:** cybersecurity, malicious URL, machine learning, classification, instance selection

## Abstract

Amid the rapid proliferation of thousands of new websites daily, distinguishing safe ones from potentially harmful ones has become an increasingly complex task. These websites often collect user data, and, without adequate cybersecurity measures such as the efficient detection and classification of malicious URLs, users’ sensitive information could be compromised. This study aims to develop models based on machine learning algorithms for the efficient identification and classification of malicious URLs, contributing to enhanced cybersecurity. Within this context, this study leverages support vector machines (SVMs), random forests (RFs), decision trees (DTs), and k-nearest neighbors (KNNs) in combination with Bayesian optimization to accurately classify URLs. To improve computational efficiency, instance selection methods are employed, including data reduction based on locality-sensitive hashing (DRLSH), border point extraction based on locality-sensitive hashing (BPLSH), and random selection. The results show the effectiveness of RFs in delivering high precision, recall, and F1 scores, with SVMs also providing competitive performance at the expense of increased training time. The results also emphasize the substantial impact of the instance selection method on the performance of these models, indicating its significance in the machine learning pipeline for malicious URL classification.

## 1. Introduction

Thousands of new websites are created every day that collect user data through login functions. The large number of networks makes it challenging to determine which websites are safe and reliable [1]. In this context, the role of cybersecurity becomes critical. Cybersecurity can be defined as a set of tools or techniques aimed at protecting companies and users from cyberattacks [2]. In this vast digital landscape, malicious URLs, which are hyperlinks, stand as a prime tool that is exploited by cybercriminals to manipulate Internet users to give out sensitive and personal information. By interacting with these links, users expose themselves to consequences, from compromising sensitive information to becoming prime targets for cyberattacks.

There are various methods employed by cybercriminals to exploit the vulnerabilities of both users and systems. One of the most common methods is *phishing*, where attackers try to trick recipients into revealing sensitive information, which can have serious consequences [3]. *Defacement* is another technique used by attackers to manipulate the content of web pages by changing the underlying code. This form of cyber intrusion is frequently leveraged to undermine an organization’s website [4]. *Malware* techniques in malicious URLs are the methods that cybercriminals use to distribute and execute malware through deceptive website addresses. These techniques aim to exploit vulnerabilities in software, deceive users, and deliver harmful payloads. A report from the RSA [5] in 2013 showed that nearly 450,000 websites fell victim to phishing attacks, causing a loss of USD 5.9 billion. To counter such threats, blacklists consisting of known malicious URLs are employed. However, their efficacy remains limited due to the constant emergence of new malicious URLs associated with spam and phishing activities.

Machine learning is important in identifying known and new malicious URLs [6]. Machine learning is a mechanism by which computers are trained to interpret data, enabling them to autonomously make predictions or decisions. The most commonly used machine learning technique for identifying malicious URLs is *classification*, a branch of supervised machine learning [7]. This approach relies on previously established information to guide the learning process. Various specific machine learning models fall under this category, such as support vector machines (SVMs), decision trees (DTs), k-nearest neighbors (KNNs), and random forests (RFs). However, the efficiency of these models can be hampered when dealing with large datasets, which can significantly slow down the training process. In this context, instance selection can be used to overcome this challenge. This approach refines the dataset by choosing a smaller-yet-indicative sample, significantly saving computational resources. As a result, the learning process is expedited without compromising the essential predictive capabilities, ensuring the efficient and precise identification of malicious URLs.

This study aims to develop models based on machine learning for the accurate classification of malicious URLs. To achieve this, we applied and evaluated the performance of specific machine learning algorithms, namely, SVMs, DTs, KNNs, and RFs. Furthermore, we examined the role of instance selection methods—including data reduction based on locality-sensitive hashing (DRLSH) [8], border point extraction based on locality-sensitive hashing (BPLSH) [9], and random selection—in the speed and accuracy of the learning process for malicious URLs’ detection.

The remainder of this study is structured as follows. Section 2 gives an overview of the relevant literature about the identification of malicious URLs using machine learning. Section 3 overviews the study’s methodology, including data collection, machine learning models, and the instance selection methods employed. In Section 4, the study’s results are presented, and the performances of the models and instance selection methods are analyzed and discussed. Lastly, Section 5 presents the conclusions and the proposed directions for future work.

## 2. Related Work

Several studies were conducted on malicious URL detection and classification using various machine learning methods. These studies contributed to understanding the effective approaches and techniques for identifying and classifying malicious URLs.

In a study conducted by Li et al. [10], a dataset comprising approximately 52,000 URLs was analyzed. This dataset was divided so that 70% was used for model training, and the remaining 30% was used for validation and testing. The analysis relied on eight key input features, including the HTTPS status, the IP address, the number of dots in the domain name, and some features related to the top-level domain. Various machine learning methods were implemented in this study, including SVMs, KNNs, DTs, RFs, gradient boosting decision trees, XGBoost (XGB), and LightGBM (LGB). The results showed that the SVM model performs notably well, achieving an accuracy rate of 94.45%.

Saleem Raja et al. [11] used a dataset of about 66,000 URLs, in which 70% of the samples were devoted to model training, and 30% were used for validation and testing. The trained models included SVMs, logistic regression (LR), KNNs, Naive Bayes (NB), and RFs. About 20 features were used in the analysis, such as the URL length, HTTPS status, number of digits, alphabetic characters, and symbols in URLs. The RF model showed the highest performance, achieving an accuracy of 99%, closely followed by the SVM model, which obtained an accuracy of 98%.

In a study by Mrad [12], a large dataset was collected, encompassing different categories, including 428,103 benign URLs, 96,457 defacement URLs, 94,111 phishing URLs, and 32,520 malware samples. Various machine learning methods were implemented, including DTs, RFs, KNNs, NB, and Ada-boost. Among them, the DTs and RFs demonstrated superior performance, achieving an accuracy of 91%.

Aljabri et al. [13] used several machine learning models, such as NB, RFs, long short-term memory, and a convolutional neural network for malicious URLs’ detection. A training dataset with 1.2 million samples and a test dataset with 0.364 million records were employed. Various attributes such as the URL, IP address, JavaScript code, obfuscated code, geographical location, top-level domain, and HTTPS were considered for the classification. The training set exhibited a class imbalance, with most samples being benign, and a minority being malicious. Randomized undersampling was applied to balance the classes, resulting in a training dataset with 54,506 records. Feature selection techniques were also applied to select the most relevant features. The results showed that the NB model has the best performance, achieving an accuracy of 95%.

Ahammad et al. [3] used a dataset of 3000 URLs to identify malicious URLs, where 1500 samples were malicious, and 1500 samples were benign. The dataset had fifteen features, including the domain name, URL length, and HTTPS status. Some models based on the RF, DT, LGB, LR, and SVM methods were developed. Then, 80% of the samples were used for training and validation, and 20% of the samples were used for testing. LGB demonstrated the highest testing accuracy among the evaluated models: 86%. This was followed by RFs and DTs, with accuracies of 85.3% and 85%, respectively, while SVMs displayed the lowest testing accuracy: 83.5%.

Upendra Shetty et al. [14] utilized XGB, LGB, and RFs to detect and classify malicious URLs. A dataset of 651,191 URLs, categorized into four groups, malware, defacement, benign, and phishing, was used. A feature selection process was incorporated to enhance the decision-making capacity of the models, leading to the identification of the IP addresses and URL length and the calculation of the character/digit ratio and the non-alphanumeric characters in the URLs as the most important features. The results showed that the RF model outperforms the others, achieving an accuracy rate of over 91%.

The related work underscores the promising potential and continued relevance of employing machine learning techniques such as SVMs, KNNs, DTs, and RFs for classifying malicious URLs. Compared to the reviewed work, this study also focuses on using instance selection methods to enhance the efficiency of the training processes. In doing so, it responds to an emerging need in the field to find innovative approaches that can quickly adapt to the rapidly evolving landscape of cyber threats.

The proposed approach stands out by addressing the issue of computational efficiency through the application of instance selection methods. It provides a comprehensive analysis of the model performance, feature importance, and category-specific challenges, offering valuable insights for improving malicious URL detection and classification. The emphasis on computational efficiency and instance selection methods is a notable improvement in the field, as it directly addresses a practical challenge in cybersecurity.

## 3. Methodology

**Figure 1:**
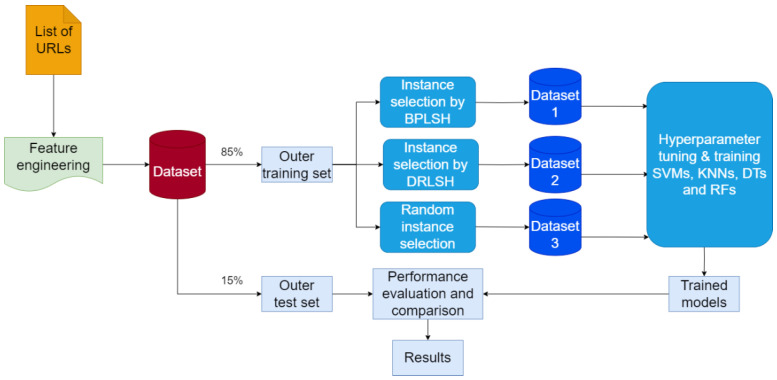
Workflow of the study.

This section presents the methodology for developing machine learning models to identify malicious URLs. It delineates the research framework, explaining the strategies and techniques utilized. Figure 1 shows the workflow of the methodology in this study. It consists of three main steps.

The initial step entailed data collection and preparation. This step involved gathering a multitude of URLs and their corresponding labels and indicating their malicious or benign status. Moreover, null values in the dataset were eliminated to maintain data integrity. Next, features (attributes) instrumental for identifying malicious URLs were extracted. The result was a dataset wherein each row represented a unique URL delineated by its extracted features and an associated output label indicating its classification. The second phase focused on developing models using SVMs, KNNs, DTs, and RFs to accurately distinguish malicious URLs. The prepared dataset was divided into an outer training set and an outer test set. The outer training set played a pivotal role in refining the hyperparameters of each model and in the subsequent training process, using the optimized hyperparameters. Various instance selection techniques were also employed to understand their influence on the model’s performance. These techniques helped generate smaller, representative subsets of the dataset, accelerating the training process. In the final phase, the performance of the generated models was compared and evaluated on the outer test set. All the steps of the workflow were implemented in MATLAB 2022. In what follows, each step is elaborated in more detail.

### 3.1. Data Collection and Preparation

**Figure 2:**
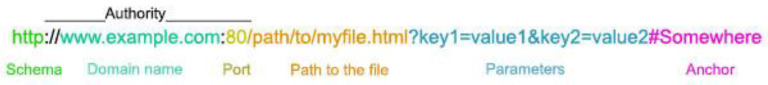
URL anatomy.

A list of 650,000 URLs, along with their respective categories—phishing, defacement, malware, and benign—was collected from the Kaggle repository (www.kaggle.com, accessed on 26 February 2023). A URL is a unique address identifying a resource, such as an HTML page. A URL consists of several parts, as shown in Figure 2. First, it is planned to specify the protocol that is used to retrieve the object, with HTTPS (encrypted connection) and HTTP (unencrypted) being the most-used protocols. Second, the IP address indicates the web server being requested, and the port indicates the gateway that should be used to access the content. The third part of a URL is the path to the object. The fourth part is a list of parameters that can be used to specify keys and values that allow other actions to be performed. Finally, an anchor allows for jumping to a specific section of the web page. Parameters and anchors may sometimes be excluded from a URL.

Raw URL data alone are insufficient for the identification of malignant URLs, as they fail to offer insightful details about the characteristics of the URLs that could aid in their classification. To bridge this gap, feature extraction becomes necessary, transforming raw URL data into quantifiable indicators that machine learning algorithms can effectively process. Hence, a total of 16 distinct input features were selected to train and develop machine learning models. These features captured crucial information needed for the classification task. The extracted input features were as follows:

- URL_length: The length of a given URL.
- Domain_length: The length of the domain name.
- Has_ipv4: Verifying the presence of an IPv4 address in each URL and subsequently returning either 1 or 0.
- Has_http: Verifying the presence of the HTTP protocol in each URL and subsequently returning either 1 or 0.
- Has_https: Verifying the presence of the HTTPS protocol in each URL and subsequently returning either 1 or 0.
- Count_dots: The number of dots.
- Count_dashes: The number of dashes.
- Count_underscores: The number of underscores.
- Count_slashes: The number of slashes.
- Count_ques: The number of question marks.
- Count_non_alphanumeric: The number of non-alphanumeric characters.
- Count_digits: The number of digits.
- Count_letters: The number of letters.
- Count_params: The number of parameters.
- Has_php: Verifying the presence of the word “php” in each URL and subsequently returning either 1 or 0.
- Has_html: Verifying the presence of the word “html” in each URL and subsequently returning either 1 or 0.

The category of each URL—phishing, defacement, malware, or benign—was used as the output feature. Hence, the resulting dataset comprised 650,000 instances, with each characterized by 16 input features and a corresponding output feature. To construct and evaluate the machine learning models, a strategic division of the dataset was implemented. Specifically, 85% of the dataset (552,500 instances) was dedicated to training the models, and the remaining 15% (97,500 instances) was reserved for testing purposes, allowing for an accurate assessment of the models’ ability to classify URLs.

### 3.2. Developing Machine Learning Models

While the prepared large training dataset (552,500 instances) provided a rich foundation for analysis, it could lead to the issue of computational inefficiency. To address this issue and ensure that the machine learning methods effectively and efficiently learn, instance selection was utilized. Instance selection methods enhance the efficiency of computations by selecting a small subset of instances representative of the original set. Many instance selection methods have been proposed, owing to the increasing number of records in datasets. In this study, we used BPLSH [9], DRLSH [8], and random selection to reduce the number of samples in the dataset.

Both DRLSH and BPLSH are based on locality-sensitive hashing. They pinpoint critical samples by scrutinizing the similarity in data points and their respective labels. While DRLSH’s main objective is to detect similar instances within each class, BPLSH expands this objective to include both similar and border instances. In DRLSH, sample reduction is achieved by independently removing nearly identical data points within each class. BPLSH, however, takes a more comprehensive approach, collectively evaluating samples from all classes. It discards similar samples only when they are distanced from the decision boundaries. The time complexity of DRLSH is less than that of BPLSH. In random selection, a small subset of instances in a random manner is selected to form a reduced training dataset. Simplicity and low computational complexity are the biggest advantages of such a method, making it feasible for many different types of datasets. By applying the instance selection methods, three datasets were derived.

In the study’s dataset, comprising four categories, benign, defacement, phishing, and malware, the BPLSH method focused on analyzing the boundaries that separate these categories. In contrast, DRLSH concentrated on the examination of similar samples within the dataset. Meanwhile, random selection reduced the dataset’s size by using the randperm function.

The use of these instance selection methods, BPLSH, DRLSH, and random selection, differed from other techniques due to their varying selection criteria, objectives, and handling of decision boundaries. These distinctions made each method suitable for particular contexts and dataset characteristics.

In this study, we employed machine learning models for the detection of malicious URLs. Symbolic AI can also be used as an alternative approach to detect malicious URLs by defining a set of rules or patterns that characterize such URLs. Symbolic AI is well-suited for well-defined, rule-based tasks like identifying known patterns of malicious URLs. However, it encounters challenges when it comes to adapting to new and evolving threats.

In contrast, machine learning models, especially when complemented with instance selection methods such as BPLSH, DRLSH, and random selection, offer greater flexibility, scalability, and adaptability to address the dynamic nature of evolving threats. These models can autonomously learn and adapt to new patterns and variations in malicious URLs, making them a preferred choice for modern URL threat detection systems.

In this study, the pressing challenge was prolonged machine learning model training times. To address this, we implemented instance selection methods. These techniques, by reducing dataset size, expedited training, facilitated efficient pattern discovery in the data, and enhanced research efficiency.

After instance selection, four machine learning models, namely, DT, RF, KNNs, and SVM, were developed to identify malicious URLs. These models were selected due to their widespread popularity and extensive applications in classification. Moreover, the performance of these models was further enhanced by employing Bayesian optimization for hyperparameter tuning, a process that allows for fine-tuning the model configurations to achieve the best possible results.

#### 3.2.1. DTs

A DT is a machine learning algorithm that makes decisions based on a tree-like model of decisions. It starts with a single node, representing the entire dataset, which is then partitioned based on specific conditions. This process continues recursively, creating a structure that resembles a tree with branches and leaves. Each internal node of the tree corresponds to a feature, each branch represents a decision rule, and each leaf node represents an outcome, i.e., the predicted class. DTs are favored for their interpretability and simplicity, as they mirror human decision-making processes and do not require complex computations. This approach makes it easier to identify the most important decision factors and how they affect the outcome [15].

The performance of DTs is influenced by their hyperparameters, which are essentially adjustable parameters that must be fine-tuned before the model is trained. The crucial hyperparameters of DTs are the maximum number of decision splits (branch nodes) and the splitting criterion. The maximum number of decision splits refers to the maximum number of splitting points the decision tree can create from the root to the furthest leaf. It regulates the complexity of the model, preventing overfitting by restricting the number of possible decision splits. The splitting criterion refers to the function used to measure the quality of a split. This function essentially provides a means of deciding the most effective way to separate the instances at each decision node based on their feature values. The most commonly used criteria are *Gini impurity* and *entropy*. Gini impurity is a measure that quantifies the probability of incorrect classification; a Gini impurity of 0 represents perfect purity, while a score of 1 suggests a high likelihood of misclassification. In contrast, entropy measures the level of disorder within an input set, with maximum entropy signifying that all classes are equally represented (hence, high disorder) and a score of 0 indicating total order.

#### 3.2.2. RFs

An RF is a machine learning algorithm that belongs to the ensemble learning method class. It combines multiple decision trees to create a more powerful and robust predictive model. Each tree in the forest is grown using a randomly selected subset of features and data points, preventing overfitting and enhancing the model’s generalization ability. An RF makes a final forecast by combining the predictions of individual trees, typically through a majority voting system. Unlike a single decision tree that may suffer from overfitting, the aggregation of many decision trees in an RF significantly reduces this risk [16].

In constructing and optimizing RFs, several critical hyperparameters come into play, including the number of trees, the maximum number of decision splits, the number of predictors to sample, and the splitting criterion. The number of trees determines the total number of decision trees grown in the forest. A higher number of trees generally improves the robustness and accuracy of the model, but it also increases the computational demands. The maximum number of decision splits limits the number of levels each decision tree can have. Increasing the number of decision splits enables the model to better capture complex relationships in the data but makes the model more prone to overfitting. The number of predictors to sample refers to the number of randomly selected features considered at each split in the decision tree, offering control over the model’s randomness and bias–variance balance. Lastly, the splitting criterion plays a role similar to its function in DTs, aiding in the selection of the optimal decision rule at each node.

#### 3.2.3. SVMs

An SVM classifier works by determining the hyperplane that best separates the classes of data points. This is achieved by maximizing the margin, which is the distance between the separating hyperplane and the nearest data points from both classes. The hyperplane is chosen to have the maximum margin, ensuring that it is as far away as possible from the data points of both classes. This feature ensures a greater separation between classes, thereby enhancing model accuracy. Concurrently, SVMs work toward minimizing classification errors, achieving a balance between margin maximization and error minimization for the optimal decision boundary [17].

The key hyperparameters of SVMs include the choice of the kernel function, box constraint, kernel-specific parameters, and multiclass method. The kernel is a function that transforms the data into a higher dimensional space, making it possible to find a separating hyperplane when the data are not linearly separable in their original space. The most common kernels include linear, polynomial, and Gaussian. The box constraint, a regularization parameter, controls the trade-off between the model’s complexity and its capacity to tolerate errors. Indeed, it is a controlling factor in determining the model’s sensitivity to misclassification errors during training. Lower values for this parameter make the model less sensitive to misclassified training samples, leading to a simple model but increasing the risk of underfitting. Conversely, higher parameter values push the model to reduce classification errors for the training samples, even at the expense of a narrow decision margin, increasing the risk of overfitting. The kernel-specific parameters depend on the type of kernel chosen. For instance, the Gaussian kernel has a parameter called the kernel scale that adjusts the shape of the decision boundary. Higher kernel scale values may create rigid boundaries, risking underfitting, while lower values allow for flexible boundaries that can capture complex data patterns but may cause overfitting. The multiclass method specifies the strategy used to handle multiple classes. Common approaches include one-vs-one and one-vs-rest strategies. In the one-vs-one strategy, a separate SVM model is trained for each pair of classes, while in the one-vs-rest strategy, an individual SVM model is trained for each class against all other classes.

#### 3.2.4. KNNs

KNNs are a supervised learning algorithm that is valued for its simplicity and broad applicability. KNNs first identify the *k* training instances closest to a given test instance. Then, the test instance is subsequently categorized into the most prevalent class among these *k* neighbors [18]. The principal hyperparameter for KNNs is *k*, representing the number of neighbors to consider during classification. Smaller *k* values make the model sensitive to noise, whereas larger *k* values increase computational demand. The choice of *k* is, thus, a trade-off between model stability and computational efficiency.

#### 3.2.5. Hyperparameter Tunning Using Bayesian Optimization

Optimizing hyperparameters is crucial for achieving the highest level of performance in machine learning models. While there are numerous proposed methods for automatically tuning hyperparameters [19,20], tackling the non-convex nature of hyperparameter optimization problems necessitates the usage of global optimization algorithms. In line with this, we employed Bayesian optimization in this study [21]. Bayesian optimization provides an effective and sophisticated approach to hyperparameter tuning. The effectiveness of Bayesian optimization lies in its strategic design for the global optimization of computationally expensive functions. It constructs a probability model of the objective function by using past evaluations and employing Bayesian inference and Gaussian processes. This allows the algorithm to smartly explore and exploit the hyperparameter space, thereby pinpointing the hyperparameters most likely to yield superior performance.

To evaluate the performance of different hyperparameter settings in a robust manner, we utilized *k*-fold cross-validation. This involves partitioning the training dataset into *k* equally sized subsets or folds. Each subset serves as a validation set exactly once, while the model is trained on the remaining *k* − 1 subsets. The objective function that guides Bayesian optimization toward optimal hyperparameters is the average classification accuracy obtained from these validation sets. In this way, *k*-fold cross-validation complements the Bayesian optimization procedure, providing a more reliable assessment of each hyperparameter setting’s performance.

Standard 5-fold cross-validation was used, as it strikes a balance between accurately estimating model performance and managing computational costs. Each model had specific hyperparameters configured: DTs involved maximum splits and split criterion; KNNs used 10 neighbors and squared inverse distance weight; RFs employed ensemble method, number of learners, maximum splits, and predictors to sample; SVMs featured one-vs-one multiclass method, box constraint level, kernel scale, Gaussian kernel, and standardized data. These hyperparameter settings were determined to optimize each model’s performance.

#### 3.2.6. Evaluation Metrics

The performance of the machine learning models in classifying malicious URLs was evaluated using three assessment metrics: precision, recall, and F1 score. Precision, calculated using Equation (1), is a measure that encapsulates the proportion of true positive instances within all the instances that the model classified as positive. For a specific class, e.g., malware, precision is equal to the ratio of URLs accurately identified as malware over the total URLs predicted as malware. Recall is a metric that computes the proportion of true positive instances that the model correctly identified (Equation (2)). For the malware category, as an example, recall is the ratio of URLs that are correctly classified as malware to the overall actual malware URLs. The F1 score combines precision and recall, providing a balanced measure of the two metrics in a model’s performance (Equation (3)). In Equations (1)–(3), true positives are the instances correctly predicted as positive—e.g., malware URLs correctly identified as malware. False positives are the instances that are incorrectly predicted as positive—e.g., non-malware URLs wrongly flagged as malware. False negatives are the instances incorrectly predicted as negative—e.g., malware URLs incorrectly labeled as non-malware.

(1) Precision=(True positives)/(True positives+False positives)

(2) Recall=(True positives)/(True positives+False negatives)

(3) F1 Score=2×(Precision×Recall)/(Precision+Recall)

## 4. Results and Discussion

This section presents an assessment and comparison of the performance of the machine learning models as well as the instance selection methods in classifying malicious URLs. The effectiveness of the models was evaluated using the precision, recall, and F1 score. The results provided valuable insights into the capabilities and limitations of the models, enabling a better understanding of their performance in identifying malicious URLs. All the experiments were conducted in MATLAB 2022.

**Figure 3:**
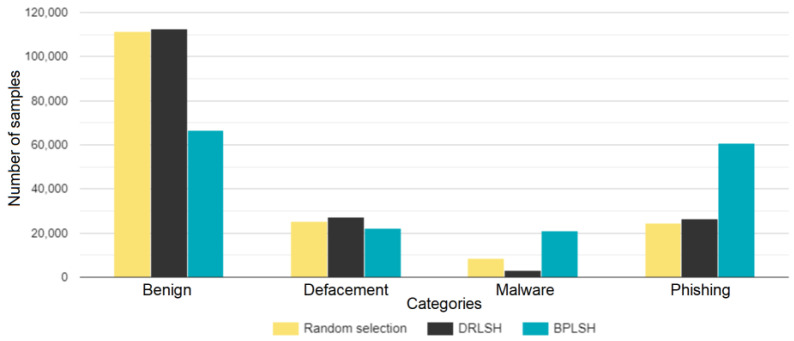
Number of samples in each obtained dataset using random selection, DRLSH, and BPLSH.

The prepared dataset was divided into two parts to ensure both efficient learning and accurate model evaluation: the outer training set, encompassing 85% of the total samples, and the outer test set, constituting the remaining 15%. Given the considerable size of the training dataset, comprising 552,500 instances, we applied instance selection (random selection, DRLSH, and BPLSH) on the outer training set to address potential computational inefficiencies. Figure 3 shows the number of samples across categories in each derived dataset. The benign category consistently holds the highest sample count for all three datasets, reaching a peak in the DRLSH dataset at 112,712. The dataset derived by BPLSH, however, displays a notably higher count of phishing samples, 60,708, compared to the datasets obtained by DRLSH and random selection. The malware category consistently has the least samples across all datasets, particularly within the DRLSH dataset at 3054. These variations underscore the different sampling techniques’ effects on the sample distributions within categories.

In the next step, the machine learning models were trained on the derived datasets. The hyperparameters of the models were tuned using Bayesian optimization in combination with *k*-fold cross-validation. The number of folds *k* was set to 5, providing a good balance between accurately estimating the model’s performance and managing the computational cost of cross-validation.

**Figure 4:**
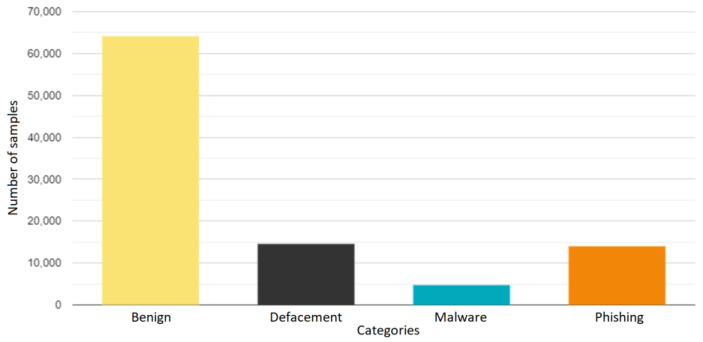
Number of samples for each category in the test dataset.

The performance of the trained models was evaluated on the outer test set, comprising approximately 97,500 samples. Figure 4 shows the number of samples for each category in the test set. As can be seen, each category has a different number of instances. This variation reflects the real-world distribution of different types of URLs, enabling a more realistic evaluation of the models’ performance in classifying various categories.

The evaluation results of the trained models on the test set in terms of precision, recall, and F1 score are shown in Table 1. Table 1 also shows the training time for each model. The effect of the instance selection methods and machine learning models on the classification of URLs is noticeable. RFs generally show a high precision, recall, and F1 score, especially when trained with randomly selected data or using the BPLSH method. In these cases, RFs notably outperform all other models. SVMs, while also demonstrating competitive performance, are hindered by considerably higher training times. DTs and KNNs show more variability in their performance across different instance selection methods. The DT model maintains moderate performance on all datasets. The KNN model, in contrast, exhibits a significant decrease in performance when trained on the BPLSH-selected dataset. Overall, the results show that the choice of instance selection method can significantly impact the performance of different machine learning models, highlighting the importance of this step in the machine learning pipeline. It also points to the necessity of striking an optimal balance between performance and computational efficiency when selecting a model.

**Figure 5:**
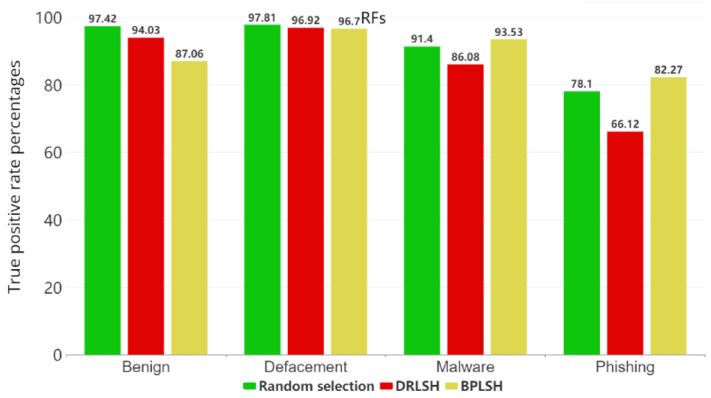
True positive rates for each category for the RF model.

**Figure 6:**
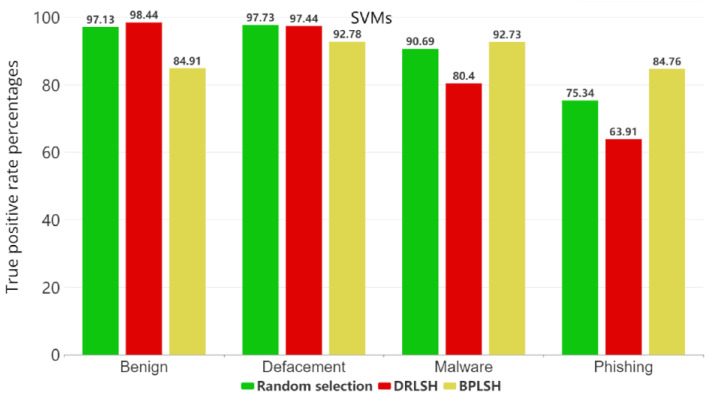
True positive rates for each category for the SVM model.

**Figure 7:**
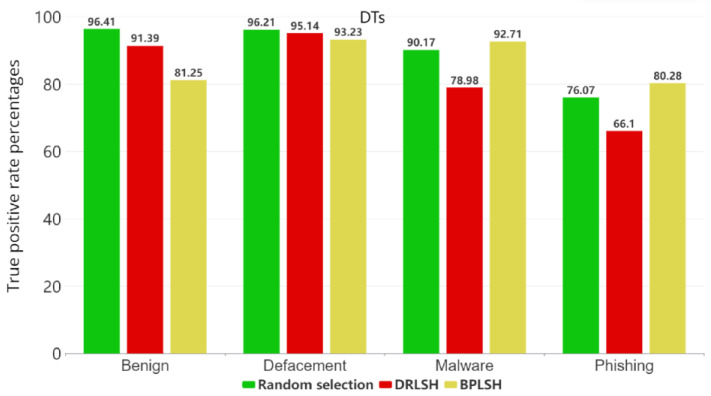
True positive rates for each category for the DT model.

**Figure 8:**
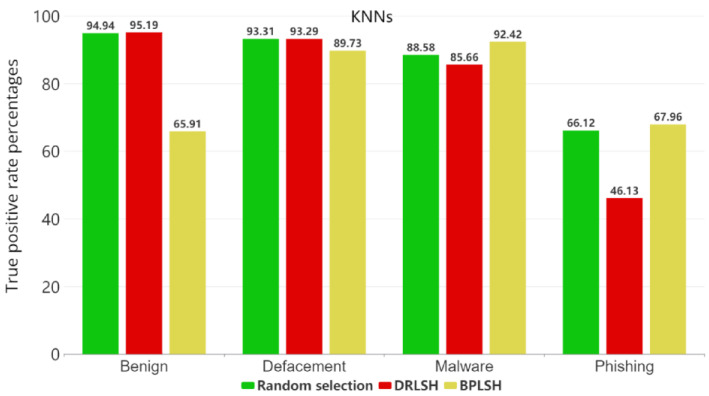
True positive rates for each category for the KNN model.

Figure 5, Figure 6, Figure 7 and Figure 8 show the true positive rates (TPR) for each category across all machine learning models and instance selection methods. The TPR show the percentage of positive instances correctly identified by the model compared to the total number of positive instances in the dataset. It is clear that classifying phishing URLs remains a challenge for all models, with consistently lower true positive rates in this category compared to others. In contrast, the defacement category is typically the best-identified category by most models, showing the highest true positive rates. While RFs and SVMs tend to maintain strong overall performance, the results vary depending on the instance selection method. The KNNs model, for example, shows a notable decrease in performance when the BPLSH method is used. The effectiveness of instance selection methods also substantially differs depending on the model and category. The results emphasize the importance of carefully selecting both the appropriate machine learning model and instance selection method to optimize the identification of malicious URLs within the cybersecurity domain.

**Figure 9:**
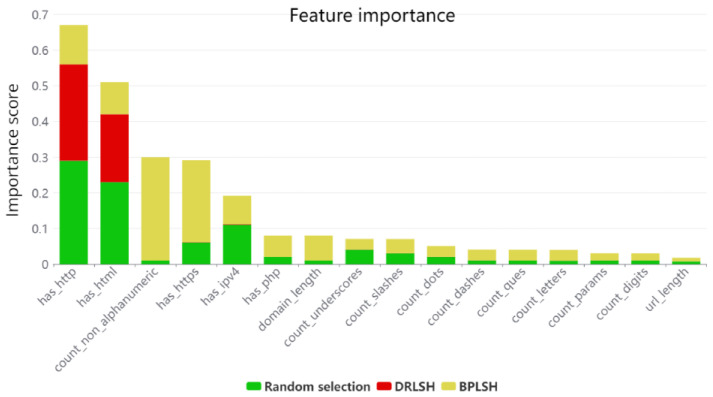
Comparative analysis of feature importance across different datasets: a visualization of each feature’s relevance in classifying malicious URLs within each dataset.

In the next step, the most important features in each dataset were selected using *minimum redundancy—maximum relevance* (MRMR) [22]. MRMR is designed to select features that not only bear a high correlation with the classification outcome (*maximizing relevance*) but also exhibit minimal intercorrelation (*minimizing redundancy*). This dual purpose is achieved using *mutual information*, a metric quantifying the information shared between variables. In MRMR, mutual information measures the *relevance* of each feature by calculating its shared information with the target variable and assesses the *redundancy* between features by examining the shared information between each pair. By maximizing relevance and minimizing redundancy, MRMR results in the selection of highly informative, non-overlapping features. Figure 9 shows the feature importance scores for all 16 features in the datasets. According to Figure 9, the importance scores for random selection and BPLSH are higher than those for DRLSH. Except for the features “has_http” and “has_html”, the scores for DRLSH are less than 0.01. The total importance score for the feature “has_http” in all datasets indicates that it plays the most crucial role in classifying malicious URLs. URLs that use HTTP as a protocol are not encrypted and have a high probability of being malicious.

## 5. Conclusions and Future Work

Identifying and blocking malicious URLs can enhance cybersecurity and protect users from cyber threats. This, in turn, can reduce the risk of financial harm and promote a stable and sustainable economy. In this study, we developed and evaluated four machine learning models, namely, DTs, RFs, SVMs, and KNNs, for classifying malicious URLs. To address the issue of computational inefficiency in training the models, three instance selection methods—random selection, DRLSH, and BPLSH—were applied. These techniques generated three considerably smaller but representative datasets that expedited the training process, ensuring computational efficiency. Additionally, applying the MRMR algorithm aided in highlighting the most critical features in the datasets. These findings provide valuable insights for cybersecurity practitioners, emphasizing the importance of tailored model selection and the ongoing need for innovative approaches in the fight against cyber threats.

The evaluation results revealed noticeable differences in the performance of the machine learning models. RFs generally delivered high precision, recall, and F1 scores, thereby demonstrating superior performance. SVMs also offered competitive performance, albeit at the cost of higher training times. DTs and KNNs exhibited more varied performance. While DTs maintained moderate performance, KNNs showed a significant drop in performance when trained on the BPLSH-selected dataset. These results highlighted the significance of the choice of machine learning model in achieving an optimal balance between performance and computational efficiency. Performance differences across the URL categories were also noted, with phishing URLs consistently posing a challenge for all models, whereas the defacement category showed a notably higher rate of accurate identification.

The three evaluated instance selection methods had distinct effects on model performance. Models trained with data selected via the BPLSH method generally performed well but caused a performance decrease in KNNs. On the other hand, all models trained with randomly selected data showed high performance, with RFs demonstrating high classification accuracies. These findings underscored the importance of an informed choice of instance selection method, as it significantly affects the overall effectiveness of the model in classifying malicious URLs. The total importance score of the “has_http” feature for all three instance selection methods showed that it plays an important role in identifying malicious URLs. Its high score indicated that it greatly helps in accurately classifying URLs.

To enhance the study, some improvements can be offered for future studies. Firstly, expanding the range of models under examination to encompass neural networks, NB, XGB, and LGB would be advantageous. This diversification allows for a more thorough exploration of alternative modeling approaches that could potentially outperform the existing methods at the crucial task of malicious URL identification. Secondly, to bolster the study’s relevance and effectiveness in the realm of cybersecurity, the incorporation of additional categories of malicious URLs, such as redirect URLs, scam URLs, clickbait URLs, and drive-by download URLs, is recommended. This expansion in categorization aligns with the evolving landscape of cyber threats and facilitates the development of more adaptable and robust defense mechanisms. Lastly, future research could significantly benefit from integrating larger and more diverse datasets, encompassing URLs from various sources and contexts. Such dataset enrichment not only strengthens model performance but also enhances this study’s practical applicability. These thoughtful directions for future investigations hold the potential to advance the field of malicious URL detection and classification while effectively addressing the limitations inherent in the current study.

## Figures and Tables

**Table 1:** Model’s training time, precision, recall, and F1 score in each dataset obtained using random selection, DRLSH, and BPLSH.

Instance Selection	Model	Training Time (s)	Precision (%)	Recall (%)	F1 Score (%)
Random	RFs	71 s	93.19%	91.19%	92.18%
Random	SVMs	10,793 s	92.29%	90.23%	91.25%
Random	DTs	16 s	90.64%	89.72%	90.18%
Random	KNNs	94 s	87.56%	85.74%	86.64%
DRLSH	RFs	82 s	83.32%	85.79%	84.54%
DRLSH	SVMs	18,390 s	92.21%	85.05%	88.49%
DRLSH	DTs	21 s	80.94%	82.88%	81.90%
DRLSH	KNNs	92 s	79.60%	80.07%	79.84%
BPLSH	RFs	75 s	86.13%	89.89%	87.97%
BPLSH	SVMs	16,681 s	82.55%	88.80%	85.56%
BPLSH	DTs	23 s	81.92%	86.87%	84.32%
BPLSH	KNNs	88 s	67.44%	79.01%	72.77%

## Data Availability

The dataset employed in this study was sourced from Kaggle and is publicly available for access and use via their website at www.kaggle.com (accessed on 26 February 2023).
